# Inequalities in lung cancer mortality trends in Brazil, 2000–2015

**DOI:** 10.1038/s41598-020-76165-4

**Published:** 2020-11-05

**Authors:** Gisele Aparecida Fernandes, Fabrício dos Santos Menezes, Luiz Felipe Silva, José Leopoldo Ferreira Antunes, Tatiana Natasha Toporcov

**Affiliations:** 1 Department Epidemiology, School of Public Health, University of São Paulo, São Paulo, SP Brazil; 2 Department of Health Education, Federal University of Sergipe, Lagarto, SE Brazil; 3 Institute of Natural Resources, Federal University of Itajubá, Itajubá, MG Brazil; 4 Group of Epidemiology and Statistics on Cancer, AC Camargo Cancer Center, São Paulo, SP Brazil

**Keywords:** Lung cancer, Cancer epidemiology

## Abstract

The present study was conducted to evaluate the socioeconomic inequality related to lung cancer mortality rates and trends between 2000 and 2015 according to gender in Brazil. We retrieved the death records for cases of lung cancer (ICD-10 C33 C34) from 2000 to 2015 in adults age 30 years and older in Brazilian Regions from official databases (DATASUS) and corrected for ill-defined causes. The Prais-Winsten regression method and Pearson correlation were applied. The results were considered statistically significant when p < 0.05. The correlation between the lung cancer mortality rates and the HDI decreased when the rates for the first and last years of the historical series were compared in men (r = 0.77; r = 0.58) and women (r = 0.64; r = 0.41). However, the correlation between the trends in the lung cancer mortality rates and the HDI was negative in men (r = − 0.76) and women (r = − 0.58), indicating larger reductions (or smaller additions) among the Federative Units with the highest HDI, in contrast to trends reflecting a greater increase in those with the lowest HDI. Our results suggest a relevant inequality in the trends of mortality from lung cancer in Brazil.

## Introduction

Lung cancer is the leading cause of cancer death, accounting for 1.7 million deaths worldwide^1^ and 26,498 deaths in Brazil in 2015^2^. Approximately 58% of lung cancer cases occur in less developed countries^3^.

Although smoking is acknowledged to be the main risk factor for lung cancer in the twentieth century^4,5^, the occurrence of the disease is also associated with air pollution, occupational exposures, socioeconomic status, genetic factors, radiation and diet^6^.

Behavioral and environmental factors seem to influence lung cancer carcinogenesis^7^. According to the Global Burden of Diseases, more developed regions, such as the United States and Europe, show higher lung cancer mortality rates^8^. In Brazil, previous studies described higher disease mortality rates in the southern region^9,10^. A study conducted in the city of São Paulo found that mortality from this neoplasm is associated with socioeconomic factors^7^.

In contrast, previous studies reported larger reductions (or smaller increases) in lung cancer mortality rates in regions with a highest human development index (HDI)^11,12^, suggesting an unfavorable scenario. In Brazil, reductions in the lung cancer mortality rates in men and increases in women were reported, with heterogeneity in the rates according to region^9,10^. However, we did not find evidence of inequalities, as assessed by means of the relationship between human development in the various regions and trends in lung cancer mortality in the country. Little is known about the socioeconomic inequalities in lung cancer mortality rates among the Federative Units of Brazil or about the changes that have occurred over time. Identifying inequalities in mortality trends, and not just their magnitude, is important for identifying vulnerable populations.

Although Brazil has a successful history of decreased tobacco prevalence (GBD, 2017), due to the 1989 launch of the national tobacco control program^13,14^, studies are needed to evaluate the effectiveness of this public policy, particularly if this policy has managed to equitably reduce lung cancer mortality rates across the country.

This study assessed the socioeconomic inequality related to the rates and trends in mortality from tracheal, bronchial and lung cancer between 2000 and 2015 according to gender in Brazil.

## Methods

We conducted a time-series study using lung cancer mortality information and population estimates for the Federative Units and Brazil between 2000 and 2015. Mortality data were extracted from the Ministry of Health Mortality Information System (SIM), provided by the Unified Health System Department of Informatics (Datasus)^15^. Population data were obtained from the Datasus website, which provides estimates from the Interagency Health Information Network (RIPSA) at the expense of the Brazilian Institute of Geography and Statistics (IBGE)^16^. HDI data were extracted from the United Nations Development Program (UNDP)^17^. Records with missing information about gender and age (0.06%) were excluded from the analysis.

Age-standardized mortality rates across the Federative Units and for Brazil were calculated for each of the 16 years in the series for both genders and for age groups 30 and older. Age-standardization was performed using the direct method with the standard world population modified by Doll^18^.

We included deaths from tracheal, bronchial and lung cancer, codes C33 and C34 of the International Classification of Diseases, 10th edition (ICD-10). Proportional redistribution was applied to correct for unknown, incomplete or poorly defined diagnostic categories. This technique consists of proportionally redistributing the deaths coded in each chapter of the ICD-10 among the deaths from specified causes, according to age groups and excluding external causes. For example, all deaths from unspecified upper respiratory tract cancer were redistributed according to age, year, gender, and Federation Unit among deaths from tracheal, bronchial and lung cancer, according to the proportion of deaths from this cause among the total number of deaths with specified causes. However, considering that cancer death records are often more satisfactorily reported than other causes of death (due to their chronic nature and demand for medical and hospital care), and to avoid overestimation of the number of cases, lung cancer was attributed to 50% of the calculated weight corresponding to neoplasms in each gender, age, year, Federation Unit and cancer type group^19^. The process consisted of 3 steps:

(1) Redistribution of deaths certified as unspecified upper respiratory tract cancer (C39) according to the proportion of lung cancer deaths originally certified among cancers of the respiratory tract and intrathoracic organs (C30–C39); (2) Redistribution of cancer deaths with incomplete diagnoses (C76 to C80 and C97) according to the proportion of lung cancer deaths among deaths from all cancers, except nonmelanoma skin cancer; (3) Redistribution of deaths from ill-defined causes (R00–R99) according to the proportion of lung cancer deaths among all-cause deaths, except external causes, multiplied by 50%^19^.

After the death correction process, in each age, year and gender group, all deaths identified as resulting from bronchial and lung tracheal cancer (ICD-10 C33–C34) were added.

The trend estimates followed the methodological guidelines presented by Antunes and Waldman^20^. Annual percent change (APC) was calculated using Prais-Winsten generalized linear regression, which predicts first-order autocorrelation correction in time-series analysis. This procedure allowed for the classification of mortality rates as either increasing, decreasing or stable and for the quantification of the annual average increase or decrease in the rates and their respective 95% confidence intervals.

To examine the changes in lung cancer mortality-related inequality, we performed two distinct analyses: (1) correlation between the HDI and age-standardized average lung cancer mortality rate in the first 5 (2000–2004) and the last 5 years (2011–2015) of the historical series, considering that the lower the correlation is, the lower the inequality is; and (2) correlation between the HDI and APC, considering that the lower the correlation is, the lower the inequality in the rate trend is. Both analyses used the Pearson correlation test. The correlation and trend analyses were conducted using STATA 14.1 (College Station, Texas, 2015), and a p-value < 0.05 was considered significant. Tabwin^21^ software was used to plot thematic maps to better visualize the results using the quartiles of mortality rates to represent the description of mortality. The research ethics committee of the School of Public Health of the University of São Paulo approved this study under the protocol number 2.518.202 in 2018.

## Results

In total, 230,933 lung cancer deaths were reported in men, and 106,526 lung cancer deaths were reported in women, between 2000 and 2015 in Brazil. Men had a higher average age-standardized mortality rate (38.43 per 100,000 men) compared to women (16.77) at 16 years of follow-up. There was a declining trend in the mortality rate among men and an increasing trend in women.

**Figure 1:**
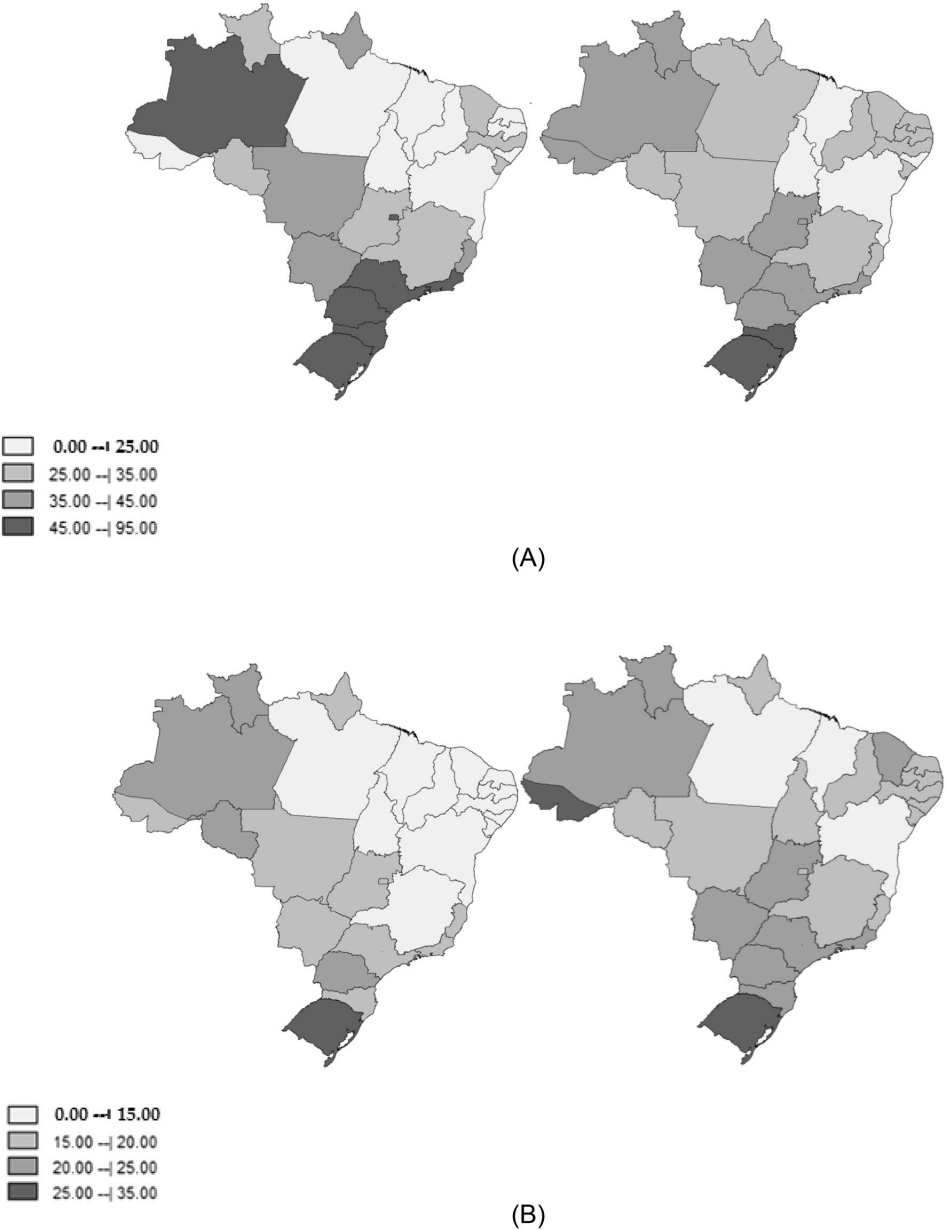
Average age-standardized mortality rates for lung cancer per 100,000 population in the first 5 years of the historical series (left) and the last five years of the historical series (right): **(A)** male; **(B)** female. Federative Units of Brazil, 2000—2015.

The distribution of the rates by Federative Units showed that lung cancer mortality in both genders was often higher in those with a higher HDI compared to those with a lower HDI. At the end of the study period (between 2011 and 2015), among men, the highest rates were observed in Rio Grande do Sul, Santa Catarina, Acre, and Rio de Janeiro, and the lowest were observed in Alagoas, Maranhão, Bahia and Tocantins. Among women, the highest rates were in Rio Grande do Sul, Acre, Santa Catarina, and Amazonas, and the lowest were in Bahia, Maranhão, Pará and Tocantins (Table 1; Fig. 1).

**Table 1:** Age-standardized mortality rates for tracheal, bronchial and lung cancer and annual percentage variation.

	HDI 2010	Male			Female		
		ASR		APC (CI 95%)	ASR		APC (CI 95%)
		2000–2004	2011–2015		2000–2004	2011–2015	
**North**							
Acre	0.663	22.94	42.92	4.56 (− 0.90; 10.33)	17.33	27.98	**6.40 (3.14; 9.75)**
Amapá	0.708	40.33	34.32	− 1.62 (− 3.38; 0.18)	17.86	15.73	3.03 (− 5.54; 12.37)
Amazonas	0.674	45.59	41.42	**− 0.91 (− 1.71; − 0.11)**	22.86	24.08	0.12 (− 1.04; 1.29)
Pará	0.646	23.63	25.60	0.76 (− 0.05; 1.58)	11.72	13.90	**1.55 (0.30; 2.82)**
Rondônia	0.690	30.66	33.14	**1.03 (0.02; 2.05)**	21.60	19.12	− 1.03 (− 3.04; 1.02)
Roraima	0.707	27.56	37.65	2.79 (− 3.18; 9.12)	21.03	21.85	0.42 (− 4.09; 5.13)
Tocantins	0.699	21.40	24.02	1.91 (− 0.15; 4.02)	10.51	15.15	**3.67 (2.00; 5.37)**
**Northeast**							
Alagoas	0.631	16.97	20.58	1.86 (− 1.37; 5.20)	11.59	16.07	**3.64 (0.98; 6.38)**
Bahia	0.660	18.29	21.90	**1.68 (0.94; 2.42)**	8.53	12.79	**3.85 (3.15; 4.56)**
Ceará	0.682	25.60	32.87	**2.38 (1.28; 3.49)**	14.59	23.92	**4.66 (3.99; 5.35)**
Maranhão	0.639	14.42	21.57	**4.10 (2.96; 5.25)**	6.89	13.00	**6.23 (5.11; 7.37)**
Paraíba	0.658	15.00	26.64	**4.91 (3.42; 6.43)**	7.20	18.23	**8.54 (7.28; 9.81)**
Pernambuco	0.673	27.69	32.09	**1.16 (0.36; 1.97)**	11.68	17.49	**3.63 (2.40; 4.87)**
Piauí	0.646	20.38	29.46	**3.60 (2.07; 5.15)**	8.31	15.86	**6.09 (5.3; 6.89)**
Rio Grande do Norte	0.684	22.48	29.13	3.96 (− 0.03; 8.11)	12.93	19.71	**4.00 (3.00; 5.01)**
Sergipe	0.665	28.09	28.08	0.22 (− 1.57; 2.04)	12.98	16.02	**1.93 (0.47; 3.41)**
**Center-West**							
Distrito Federal	0.824	46.98	36.07	**− 2.41 (− 3.23; − 1.59)**	19.10	19.44	0.11 (− 0.85; 1.08)
Goiás	0.735	34.16	36.75	**0.65 (0.17; 1.14)**	19.55	22.76	**1.55 (0.85; 2.26)**
Mato Grosso	0.725	36.26	34.59	− 0.46 (− 1.37; 0.46)	16.63	19.47	**1.55 (0.65; 2.46)**
Mato Grosso do Sul	0.729	39.20	38.66	0.08 (− 1.09; 1.26)	17.83	21.12	1.44 (− 1.71; 4.69)
**Southeast**							
Espírito Santo	0.740	39.49	34.86	**− 1.15 (− 1.66; − 0.64**)	15.66	16.65	**0.73 (0.06; 1.41)**
Minas Gerais	0.731	31.24	31.65	0.10 (− 0.18; 0.38)	12.81	16.13	**2.05 (1.48; 2.68)**
Rio de Janeiro	0.761	60.71	42.51	**− 3.15 (− 3.41; − 2.88)**	20.00	21.99	**0.98 (0.59; 1.38)**
São Paulo	0.783	51.55	40.14	**− 2.17 (− 2.48; − 1.87**)	17.88	21.36	**1.59 (1.06; 2.12)**
**South**							
Paraná	0.749	46.19	40.75	**− 1.08 (− 1.50; − 0.65)**	21.29	23.49	**0.93 (0.46; 1.40)**
Rio Grande do Sul	0.746	90.83	72.78	**− 1.96 (− 2.43; − 1.48)**	26.57	33.24	**1.84 (1.32; 2.37)**
Santa Catarina	0.774	69.34	58.07	**− 1.48 (− 2.14; − 0.82**)	19.64	24.54	**2.12 (1.29; 2.96)**
**Brasil**	0.727	43.19	38.43	**− 0.99 (− 1.30; − 0.67)**	13.27	16.77	**2.17 (1.98; 2.37)**

Federative Units of Brazil, 2000–2015.

*HDI* human development índex, *ASR* age standardized rate, *APC* annual percent change, *CI* confidence interval.

The Federative Units with the lowest HDI presented higher annual percentage changes in the lung cancer mortality rates for both genders compared to those with the highest HDI. Although there was a reduction in mortality from 2000 to 2015 among men in Brazil as a whole (APC = − 0.99; CI: − 1.30; − 0.67), there was an increase in practically all of the Federative Units of the Northeast and Rondônia. Among women, there was a significant increase in the country (APC = 2.17; CI: 1.98; 2.37) and stability in Rondônia, Amapá, Amazonas, Federal District, Mato Grosso do Sul and Roraima (Table 1; Fig. 1). We found some heterogeneity in trends: Most Federative Units showed increased mortality in women (21 out of 27) and reduced morality in men (8 out of 27).

**Figure 2:**
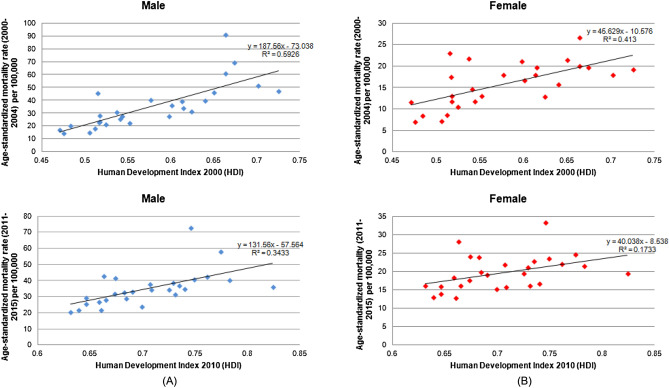
**(A)** Correlation between the average age-standardized mortality rate for lung cancer in male per 100,000 population in the first 5 years of the historical series and the last 5 years of the historical series and Human Development Index (HDI). **(B)** Correlation between the average age-standardized mortality rate for lung cancer in female per 100,000 population in the first five years of the historical series and the last 5 years of the historical series and Human Development Index (HDI). Federative Units of Brazil, 2000—2015.

The average lung cancer mortality rates in the first and last five years of the historical series positively correlated with the HDI in men (2000–2004; r2 = 0.59, r = 0.77, p < 0.001; 2011–2015; r2 = 0.34, r = 0.58, p = 0.001) and women (2000–2004; r2 = 0.41, r = 0.64, p < 0.001; 2011–2015; r2 = 0.17, r = 0.41, p = 0.030). This finding indicates higher rates among the Federative Units with the higher HDI levels. However, the correlation decreased over the historical series in both genders (Fig. 2).

**Figure 3:**
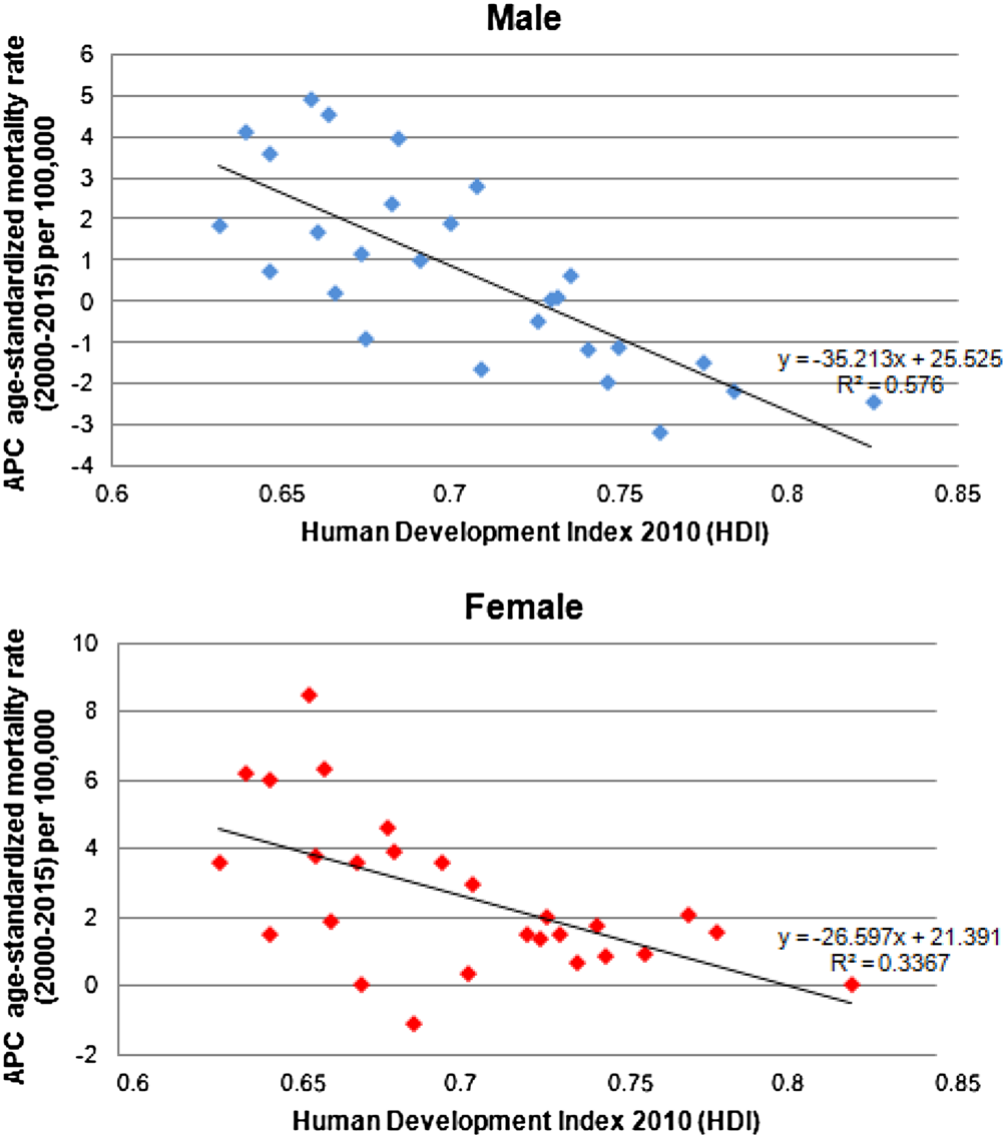
Correlation between annual percentage changes in age-standardized lung cancer mortality rates in male and female, per 100,000 population and Human Development Index (HDI). Federative Units of Brazil, 2000—2015.

The lung cancer mortality rate APCs were negatively correlated with the HDI in men (r2 = 0.57, r = − 0.76, p < 0.001) and women (r2 = 0.33, r = − 0.58, p = 0.001), indicating a greater percentage reduction in mortality rates among the Federative Units with higher HDIs (Fig. 3).

## Discussion

In the present study, we found differences in lung cancer mortality according to gender, with higher rates in men compared to women. A higher state HDI was related to higher lung cancer mortality rates, but this correlation decreased between the beginning and the end of the period. There was an increasing trend among women and a decreasing trend among men throughout Brazil, with heterogeneity among the Federation Units. The Federation Units with the highest HDI had lower values of annual variation in lung cancer mortality.

The lung cancer mortality rates were significantly higher among men compared to women, but with decreasing trends among men and increasing trends among women, in Brazil. This finding is in agreement with previous studies conducted in the country^10,22–24^ .International studies assessing worldwide lung cancer trends across five continents have also found similar results^25,26^. The increase in disease mortality among women is partly attributable to an increase in tobacco use in recent decades^22^. It is believed that, in women, the increase in smoking occurred later than it did in men. In Brazil, the increase in smoking among women was possibly associated with the affirmation of female independence and the insertion of women in the labor market and society, and this habit was stimulated by the media and movies^27^. In addition to smoking, other factors have been mentioned as relevant in the process of female lung carcinogenesis, such as physiological, hormonal and genetic mechanisms; there may also be an interaction between these mechanisms as well as with smoking^28^.

The direct correlation between the HDI and lung cancer mortality rates could be explained by the higher frequency of the exposure to risk factors, such as smoking (in previous years) and air pollution, in more developed locations^29^. It is noteworthy that the risk of death from lung cancer reflects the cumulative exposure to risk factors over previous decades^30^. Moreover, in the less developed Federative Units, mortality from preventable diseases or acute conditions could precede the occurrence of cancer, reducing its incidence and mortality. Previous studies have identified a dependency between trends in lung cancer mortality, human development, and smoking^11,12,31^.

The correlation between the HDI and lung cancer mortality rates decreased when we compared 2011–2015 with 2000–2004. This result could indicate a reduction in the inequality related to this indicator. At the end of the period (2011–2015), the correlation between the HDI and mortality rates was lower than the correlation between the HDI and mortality trends. The interpretation of this finding warrants caution, since the highest rates were initially observed among the Federative Units with the highest HDI at the beginning of the period. We found decreasing trends among the Federative Units with the higher HDIs among men and increasing or stable trends among the Federative Units with lower HDIs. A similar effect occurred among women. Ideally, the reduction in the differences between the HDI rates would be due to a decrease in mortality among those at higher risk (in this case, the Federative Units with the highest HDIs) rather than an increase in mortality among those initially at the least risk (i.e., the Federative Units with the lowest HDIs).

The assessment of the inequality in the trends showed larger reductions (or smaller increases) in the rates among the Federative Units with the higher HDIs and smaller reductions (or larger increases) in the rates among the Federative Units with the lower HDIs, which indicates the possibility of improved notification of deaths from lung cancer among those with a lower HDI. There is a need for careful monitoring by health managers to maintain health actions to prevent smoking and, consequently, lung cancer in the Federative Units with lower HDIs, where an increase in the rates was found and was more prominent. According to Antunes et al.^7^ inequalities according to gender and socioeconomic status in lung cancer mortality demonstrate evidence that the epidemiological profile of this cancer can be improved through prevention and increased access to health technologies and services.

The decline in mortality in the Federative Units with high HDIs was possibly due to the successful implementation of the national tobacco control program, which led to a decrease in smoking trends in Brazil^5,13,14^. However, this study provides evidence that the anti-smoking program may have benefited the Federative Units with greater human development, which would explain the more favorable trends observed in these places. According to the inverse equity hypothesis, public policies related to health education initially reach social groups with the highest level of human development and later are absorbed by those with the least human development^32^. In the specific case of anti-smoking policies and lung cancer, reducing the inequality in the mortality rates concomitant with the major inequality in the mortality trends is consistent with the hypothesis that there is greater benefit from smoking prevention policies in locations with high human development.

Despite a reduction in lung cancer mortality among men in Brazil, there was an increase among the Federative Units with low HDIs, such as those in the Northeast and Rondonia, while in the Federative Units with the highest HDIs, there was a declining trend in mortality rates. Similar results were found by Guerra et al^9^ in a study conducted between 1990 and 2015; however, these findings are in contrast to those from a study by Malta et al^10^, conducted between 1996 and 2011, which found a decreasing trend in lung cancer mortality in the Northeast and North.

These findings may serve as a point of reference, where preventive strategies for lung cancer should be reinforced across the country, especially in the Federative Units with low HDIs. In Brazil, for both genders, the current highest prevalence of smoking is in the population with the lowest socioeconomic level and education^33,34^ which demonstrates the vulnerability of these populations to advanced lung cancer in the next decades. Tobacco control strategies are essential in these populations, as these individuals are often targeted by the tobacco industry as a new market.

An inherent limitation of ecological studies is the impossibility of inferring causality at an individual level. However, this design allows for relevant population inferences so that our results suggest the need for special monitoring of lung cancer mortality and smoking prevalence, as well as health care, especially in the Federative Units with a lower level of human development. Another limitation is the possibility of greater detection of the basic cause of death in the Federative Units with higher HDI because of a more efficient data reporting system, more effective early detection practices and increased access to health services^35^. However, to reduce potential reporting biases, indicators were corrected for deaths from ill-defined causes.

In conclusion, the present study found a possible reduction in the relative inequality in lung cancer mortality rates. However, it was noted that this reduction was partly due to an increase in the rates of Federative Units with less human development. This finding suggests a possible improvement in the notification of deaths from lung cancer among the Federative Units with lower HDIs. This finding also suggests that the effect of smoking cessation and prevention policies in Brazil was possibly enhanced among the Federative Units with the highest HDIs, thus calling for interventions focused on the Federative Units with lower HDIs.

## Supplementary information

Supplementary Table 1.
