# Supplementary material for: Inequalities in lung cancer mortality trends in Brazil, 2000–2015
Source: Sci Rep. 2020 Nov 5;10:19164. doi: 10.1038/s41598-020-76165-4 (PMC7645723; doi:10.1038/s41598-020-76165-4)
Supplement: Supplementary file 1 — Supplementary Table 1. [file 41598_2020_76165_MOESM1_ESM.docx]

Inequalities in lung cancer mortality trends in Brazil, 2000-2015

Gisele Aparecida Fernandes, Fabrício dos Santos Menezes, Luiz Felipe Silva, José Leopoldo Ferreira Antunes, Tatiana Natasha Toporcov

Supplemental Table 1. Age-standardized mortality rates for tracheal, bronchial and lung cancer and annual percentage change. Federative Units of Brazil, 2000 – 2015

|  |  | **Male** | | | **Female** | | |
| --- | --- | --- | --- | --- | --- | --- | --- |
|  | **HDI 2010** | **ASR** | | **APC (CI95%)** | **ASR** | | **APC (CI95%)** |
|  |  | **2000-2004** | **2011-2015** |  | **2000-2004** | **2011-2015** |  |
| North |  |  |  |  |  |  |  |
| Acre | 0.663 | 10.19 | 19.04 | 4.44 (-1.06; 10.24) | 7.81 | 12.36 | **6.27 (3.14; 9.49)** |
| Amapá | 0.708 | 17.87 | 15.15 | -1.63 (-3.31; 0.08) | 7.98 | 7.01 | 3.01 (-5.50; 12.29) |
| Amazonas | 0.674 | 20.12 | 18.34 | **-0.89 (-1.68; -0.08)** | 10.12 | 10.69 | 0.15 (-0.96; 1.28) |
| Pará | 0.646 | 10.48 | 11.34 | 0.75 (-0.10; 1.60) | 5.22 | 6.15 | **1.50 (0.24; 2.78)** |
| Rondônia | 0.690 | 13.56 | 14.68 | **1.02 (0.01; 2.03)** | 9.65 | 8.49 | -1.09 (-3.10; 0.96) |
| Roraima | 0.707 | 12.13 | 16.66 | 2.88 (-3.09; 9.21) | 9.37 | 9.61 | 0.23 (-4.32; 4.99) |
| Tocantins | 0.699 | 9.47 | 10.65 | 1.86 (-0.14; 3.90) | 4.79 | 6.71 | **3.40 (1.86; 4.97)** |
| Northeast |  |  |  |  |  |  |  |
| Alagoas | 0.631 | 7.53 | 9.20 | 1.91 (-1.30; 5.23) | 5.23 | 7.20 | **3.49 (1.00; 6.05)** |
| Bahia | 0.660 | 8.11 | 9.70 | **1.67 (0.95; 2.40)** | 3.79 | 5.65 | **3.81 (3.12; 4.50)** |
| Ceará | 0.682 | 11.41 | 14.56 | **2.31 (1.24; 3.40)** | 6.51 | 10.60 | **4.60 (3.95; 5.26)** |
| Maranhão | 0.639 | 6.44 | 9.55 | **4.05 (2.87; 5.24)** | 3.08 | 5.78 | **6.24 (5.04; 7.45)** |
| Paraíba | 0.658 | 7.73 | 11.83 | **4.86 (3.42; 6.31)** | 3.24 | 8.12 | **8.46 (7.29; 9.64)** |
| Pernambuco | 0.673 | 12.24 | 14.19 | **1.16 (0.36; 1.96)** | 5.18 | 7.75 | **3.64 (2.41; 4.89)** |
| Piauí | 0.646 | 9.03 | 13.04 | **3.56 (2.00; 5.14)** | 3.72 | 7.04 | **6.00 (5.15; 6.86)** |
| Rio Grande do Norte | 0.684 | 9.94 | 12.96 | 4.01 (-0.04; 8.23) | 5.74 | 8.71 | **3.96 (2.89; 5.05)** |
| Sergipe | 0.665 | 12.51 | 12.38 | 0.10 (-1.63; 1.87) | 5.76 | 7.07 | **1.90 (0.48; 3.35)** |
| Midwest |  |  |  |  |  |  |  |
| Distrito Federal | 0.824 | 20.72 | 15.93 | **-2.40 (-3.21; -1.59)** | 8.44 | 8.58 | 0.09 (-0.88; 1.07) |
| Goiás | 0.735 | 15.06 | 16.26 | **0.69 (0.21; 1.16)** | 8.65 | 10.06 | **1.54 (0.85; 2.24)** |
| Mato Grosso | 0.725 | 16.04 | 15.33 | -0.45 (-1.33; 0.45) | 7.42 | 8.59 | **1.44 (0.54; 2.33)** |
| Mato Grosso do Sul | 0.729 | 17.32 | 17.09 | 0.09 (-1.07; 1.27) | 7.88 | 9.35 | 1.40 (-1.66; 4.56) |
| Southeast |  |  |  |  |  |  |  |
| Espírito Santo | 0.740 | 17.43 | 15.43 | **-1.14 (-1.66; -0.63**) | 7.03 | 7.37 | 0.60 (-0.05; 1.25) |
| Minas Gerais | 0.731 | 13.81 | 13.99 | 0.10 (-0.18; 0.38) | 5.68 | 7.14 | **2.01 (1.43; 2.60)** |
| Rio de Janeiro | 0.761 | 26.75 | 18.76 | **-3.13 (-3.38; -2.87)** | 8.83 | 9.74 | **1.01 (0.61; 1.42)** |
| São Paulo | 0.783 | 22.73 | 17.74 | **-2.16 (-2.46; -1.86**) | 7.90 | 9.45 | **1.60 (1.07; 2.14)** |
| South |  |  |  |  |  |  |  |
| Paraná | 0.749 | 20.41 | 17.98 | **-1.07 (-1.50; -0.65)** | 9.41 | 10.38 | 0.92 (0.46; 1.39**)** |
| Rio Grande do Sul | 0.746 | 40.01 | 32.09 | **-1.95 (-2.42; -1.48)** | 11.73 | 14.66 | **1.84 (1.30; 2.38)** |
| Santa Catarina | 0.774 | 30.58 | 25.60 | **-1.49 (-2.16; -0.81**) | 8.67 | 10.86 | **2.15 (1.31; 2.99)** |
| **Brazil** | 0.727 | 19.07 | 16.99 | **-0.98 (-1.29; -0.67)** | 7.30 | 9.09 | **1.99 (1.75; 2.24)** |

HDI: human development index

ASR: age standardized rate

APC: annual percent change

CI: confidence interval
